# The pyruvate:ferredoxin oxidoreductase of the thermophilic acetogen, *Thermoanaerobacter kivui*

**DOI:** 10.1002/2211-5463.13136

**Published:** 2021-04-04

**Authors:** Alexander Katsyv, Marie Charlotte Schoelmerich, Mirko Basen, Volker Müller

**Affiliations:** ^1^ Department of Molecular Microbiology & Bioenergetics Institute of Molecular Biosciences Johann Wolfgang Goethe University Frankfurt am Main Germany; ^2^ Present address: Department of Earth and Planetary Science Innovative Genomics Institute University of California Berkeley CA USA; ^3^ Present address: Microbiology Institute of Biological Sciences University of Rostock Rostock Germany

**Keywords:** extremophile, genetic engineering, homologous gene expression, protein production

## Abstract

Pyruvate:ferredoxin oxidoreductase (PFOR) is a key enzyme in bacterial anaerobic metabolism. Since a low‐potential ferredoxin (Fd^2−^) is used as electron carrier, PFOR allows for hydrogen evolution during heterotrophic growth as well as pyruvate synthesis during lithoautotrophic growth. The thermophilic acetogenic model bacterium *Thermoanaerobacter kivui* can use both modes of lifestyle, but the nature of the PFOR in this organism was previously unestablished. Here, we have isolated PFOR to apparent homogeneity from cells grown on glucose. Peptide mass fingerprinting revealed that it is encoded by *pfor1*. PFOR uses pyruvate as an electron donor and methylene blue (1.8 U·mg^−1^) and ferredoxin (Fd; 27.2 U·mg^−1^) as electron acceptors, and the reaction is dependent on thiamine pyrophosphate, pyruvate, coenzyme A, and Fd. The pH and temperature optima were 7.5 and 66 °C, respectively. We detected 13.6 mol of iron·mol of protein^−1^, consistent with the presence of three predicted [4Fe–4S] clusters. The ability to provide reduced Fd makes PFOR an interesting auxiliary enzyme for enzyme assays. To simplify and speed up the purification procedure, we established a protocol for homologous protein production in *T. kivui*. Therefore, *pfor1* was cloned and expressed in *T. kivui* and the encoded protein containing a genetically engineered His‐tag was purified in only two steps to apparent homogeneity. The homologously produced PFOR1 had the same properties as the enzyme from *T. kivui*. The enzyme can be used as auxiliary enzyme in enzymatic assays that require reduced Fd as electron donor, such as electron‐bifurcating enzymes, to keep a constant level of reduced Fd.

## Graphical abstract

Pyruvate:ferredoxin oxidoreductase (PFOR) is a key enzyme in anaerobic metabolism. PFOR oxidizes pyruvate to generate acetyl‐coenzyme A, carbon dioxide, and reduced ferredoxin (Fd^2−^). Reduced ferredoxin is an electron donor for many enzymes in anaerobes. Ferredoxin can be purified from some organisms and used in *in vitro* assays. When used as electron donor, it has to be kept in a reduced state by a reduction system that continuously re‐reduces ferredoxin. The ability to reduce ferredoxin makes PFOR an invaluable enzyme during *in vitro* studies. Here, we have purified PFOR from the thermophilic acetogen *Thermoanaerobacter kivui*, identified the encoding genes, and established a simple procedure for homologous production and high‐yield purification that involves affinity chromatography.

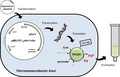

**Abbreviations**
[4Fe‐4S]: iron–sulfur cluster
CoA: coenzyme A
CODH/ACS: carbon monoxide dehydrogenase/acetyl‐CoA synthase
Ech: energy‐converting hydrogenase
Fd: ferredoxin
MALDI‐TOF: matrix‐assisted laser desorption/ionization‐time of fight
MB: methylene blue
PFOR: pyruvate:ferredoxin oxidoreductase
P‐loop: phosphate‐binding loop
Rnf: Rhodobacter nitrogen fixation
SEM: standard error of the mean
TCA: tricarboxylic acid
TPP: thiamine pyrophosphate
VIT: vacuolar iron transporter
WLP: Wood–Ljungdahl pathway

The Wood–Ljungdahl pathway (WLP) was probably the first CO_2_ fixation pathway on Earth [1] and is still employed by strictly anaerobic microorganisms: acetogenic bacteria [2], methanogenic archaea [3], and sulfate‐reducing bacteria and archaea [4]. In the WLP, 2 moles of CO_2_ are converted to the central metabolite acetyl‐coenzyme A (acetyl‐CoA) and acetogens metabolize this further to acetate [5, 6]. Most acetogens can sustain a chemolithoautotrophic lifestyle by using molecular H_2_ and/or carbon monoxide (CO) as electron donors for fixing CO_2_ in the WLP to make acetate [7]. The net ATP gain of the WLP is zero; thus, they depend on a chemiosmotic gradient for energy conservation to sustain cellular homeostasis and fuel anabolic processes [8]. The central switchpoint between catabolic and anabolic processes is acetyl‐CoA. The two enzymes that can make this key metabolite are the bifunctional carbon monoxide dehydrogenase/acetyl‐CoA synthase (CODH/ACS), which unites both branches of the WLP by fusing a methyl‐group with enzyme‐bound CO [9, 10, 11, 12, 13], and the pyruvate:ferredoxin oxidoreductase (PFOR), which can reduce and carboxylate acetyl‐CoA to pyruvate [14]. The redox potentials of the CO/CO_2_ and pyruvate/acetyl‐CoA redox pairs are, however, very low (E_0_^′^[pyruvate/acetyl‐CoA]/[CO/CO_2_] = −500/−520 mV) [15, 16], requiring an electron donor with an even lower potential. In both cases, a ferredoxin (Fd) takes on this role, whose redox potential can be as low as ~ −450 to −500 mV [16]. The produced pyruvate is then further converted via the incomplete reductive tricarboxylic acid (TCA) cycle to give rise to many different building blocks [17, 18, 19].

Most acetogens can also sustain a heterotrophic lifestyle using sugars, organic acids, or alcohols as growth substrates [20], and under these circumstances, the PFOR’s role is to provide acetyl‐CoA and low‐potential Fd^2‐^ from pyruvate. The Fd^2‐^ can then be used by energy‐converting hydrogenases (Ech) [21] or the Rnf complex to establish the chemiosmotic gradient [22], or provide electrons for a range of different soluble enzymes including Fe‐hydrogenases [23] or electron‐bifurcating enzyme complexes such as the lactate dehydrogenase (LDH/Etf) [24], the caffeyl‐CoA reductase (CarCDE) [25], the NADH‐dependent Fd^2‐^:NADP^+^ oxidoreductase (Nfn, Stn) [26, 27], several electron‐bifurcating hydrogenases [28], and the butyryl‐CoA‐dehydrogenase (Bcd‐EtfAB) [26].

The ability to provide reducing equivalents in the form of reduced Fd makes the PFOR an invaluable enzyme during *in vitro* studies of ferredoxin‐dependent enzymes. So far, most enzymatic assays requiring Fd in the reduced state rely on strong chemical reducing agents such as sodium dithionite or titanium (III) citrate. However, these reducing agents often interfere with the physiological reactions by, for example, reducing the enzyme directly. An improvement to chemical reductants was achieved when a purification protocol was established for the CODH/ACS from *Acetobacterium woodii*, which can reduce Fd with CO as reductant [29]. However, CO is a potent inhibitor of many enzymes including most hydrogenases [30], and the enzyme is highly O_2_‐sensitive [11]. Therefore, we decided to identify, purify, and characterize a PFOR that can be used to provide Fd^2‐^ in a physiological and nontoxic manner.

## Results

### Identification of potential PFOR‐encoding genes in *Thermoanaerobacter kivui*

**Fig. 1:**
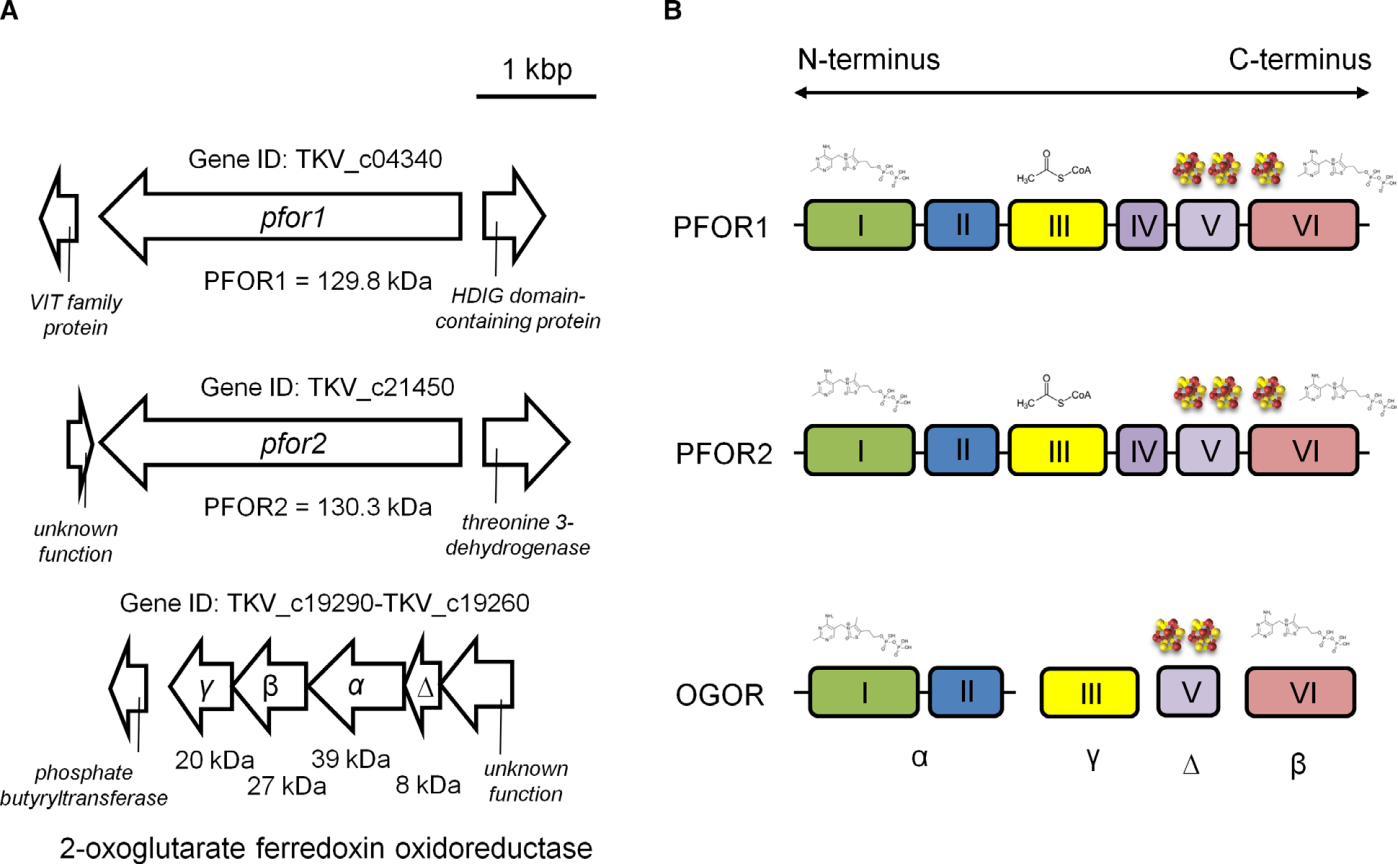
Genetic organization, architecture, and cofactors of possible PFORs of *Thermoanaerobacter kivui*. The genome of *T. kivui* encodes for three different *pfor* clusters (A). Regardless of gene arrangements, PFORs maintain a basic composition of domains I, II, III, and VI, with domain V also present in most PFORs. Domain IV is present only in dimeric PFORs. Cartoons of the domain arrangement for the catalytic units of possible PFORs in *T. kivui* are shown (B). Domains are indicated with colored boxes, with their respective domain numbers inside. Black bars connecting domains indicate that the domains are found on the same polypeptide chain. The domains that bind TPP and [4Fe–4S] cluster are indicated at the top of each domain. VIT; vacuolar iron transporter.

We used the acetogenic bacterium *T. kivui* as a model organism, a thermophilic organism that is able to sustain a lithotrophic and heterotrophic lifestyle [31]. Therefore, it must possess at least one PFOR, which functions reversibly and the enzyme should be thermostable, which is very advantageous for its potential application and storage. First, inspection of genomic data had indicated that a PFOR is encoded by the TKV_c19260‐19290 cluster [31] which would translate to proteins with molecular masses of 20.2, 27.3, 39.1, or 7.7 kDa, respectively (Fig. 1A,B). These four genes are usually fused in bacteria [32] but may retain as four separate genes in ancient complexes still found in archaea [14, 33] or hyperthermophilic bacteria [34, 35]. A closer examination of the amino acid sequence revealed that the described cluster might correspond either to the four subunits of a 2‐oxoglutarate:ferredoxin oxidoreductase (δαβγ), that catalyzes the interconversion of 2‐oxoglutarate and succinyl‐CoA in the incomplete reductive TCA cycle [1] or indeed catalyze the oxidation of pyruvate to acetyl‐CoA like in *Thermotoga maritima* [34, 35]. Oxoglutarate:ferredoxin oxidoreductase has not been demonstrated in *T. kivui*, neither has the synthesis of oxoglutarate been studied. Searching for other PFOR‐encoding genes revealed two candidate genes, *pfor1* (TKV_c04340) and *pfor2* (TKV_c21450; Fig. 1A). The amino acid sequences share 73% sequence identity with one another and the resulting protein products have predicted molecular masses of 129.8 (PFOR1) or 130.4 kDa (PFOR2; Fig. 1B). Upstream of *pfor1* lies a small gene that encodes a vacuolar iron transporter (VIT) family protein with three transmembrane helices that shows similarities to rubrerythrin. This protein is involved in an oxidative stress protection system in many anaerobes like the sulfate‐reducing bacterium *Desulfovibrio vulgaris* [36, 37]. Downstream of *pfor1* lies a putative phosphohydrolase (HDIG domain‐containing protein). Upstream of *pfor2* is a small gene encoding a protein of unknown function and downstream is a gene encoding a putative threonine 3‐dehydrogenase. Since the bioinformatic analyses did not allow a clear designation which of the two genes encode the PFOR, we attempted a purification of the PFOR based on its activity.

### Identification of pyruvate:ferredoxin oxidoreductase activity in *T. kivui* and purification of the corresponding PFOR

To investigate whether *T. kivui* has PFOR activity, initially, an enzyme assay had to be established to monitor this activity. Besides ferredoxin (Fd), isolated from *Clostridium pasteurianum* [56], methylene blue (MB) proved to be a suitable electron acceptor for the reaction. This artificial one‐electron acceptor has a E_0_^′^ of +11 mV and turns from blue in the oxidized form to colorless in the reduced state [38]. A pyruvate:MB oxidoreductase activity in an assay containing cell‐free extract of glucose‐grown cells could be observed with 20 ± 3.2 mU·mg^−1^ (using 50 µm MB), while PFOR activity was 553 ± 26.9 mU·mg^−1^ with 30 µm Fd as electron acceptor. Thus, the pyruvate:MB activity assay was used to screen for the presence of the PFOR, but further determination of the purification success and characterization of the enzyme were carried out using the physiological PFOR activity. To purify the PFOR, cell‐free extract of *T. kivui*, grown on glucose to the late exponential growth phase, was prepared. The cell‐free extract was separated into membranes and cytoplasm and the PFOR was purified from the cytoplasm by ion exchange chromatography on Q‐Sepharose, hydrophobic interaction chromatography on Phenyl‐Sepharose followed by a size exclusion chromatography on Superdex 200. Using this procedure, the enzyme was purified 50‐fold to apparent homogeneity with an average specific PFOR activity of 27.2 ± 4.1 U·mg^−1^ or pyruvate:MB oxidoreductase activity of 1.8 ± 0.3 U·mg^−1^ and a yield of 0.8 mg (Table 1).

**Table 1:** Purification of PFOR1 from *Thermoanaerobacter kivui*.

Purification step	Protein (mg)	PFOR activity^a^ (U)	PFOR activity (U·mL^−1^)	PFOR activity (U·mg^−1^)	Purification (‐fold)	Yield (%)
Cell‐free extract	1152	645	13.4	0.54	1	100
Cytoplasm	1035	577	12.8	0.56	1	89.5
Q‐Sepharose	99	212	8.5	2.1	3.9	32.9
Phenyl‐Sepharose	6.4	49	4	7.6	14.1	7.6
Superdex 200	0.8	23	45.8	27.2	50.4	3.6

^a^ PFOR activity was measured with pyruvate as electron donor and ferredoxin as electron acceptor

**Fig. 2:**
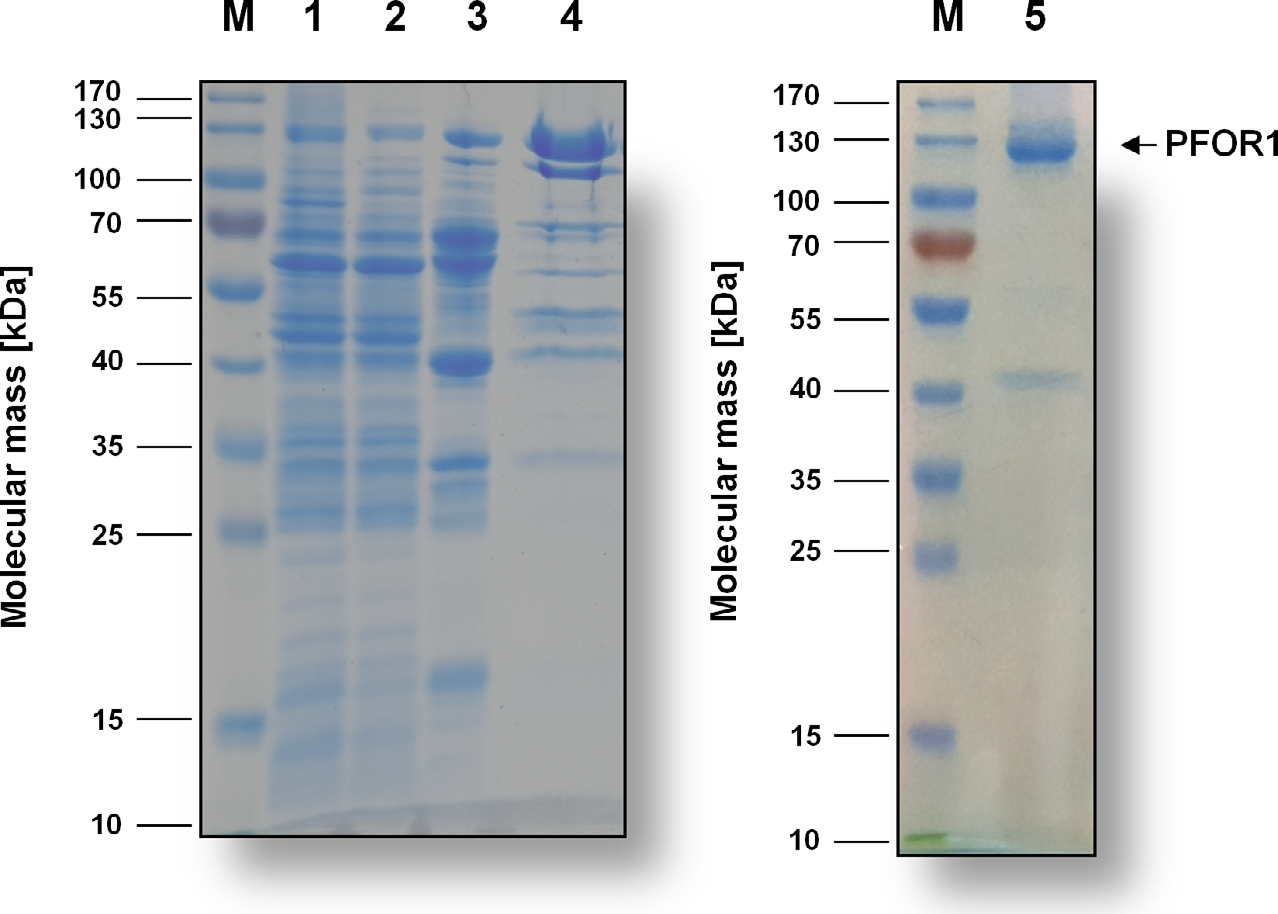
SDS/PAGE monitoring the purification process of PFOR1. Samples from the different purification steps were separated by SDS/PAGE, and proteins were stained with Coomassie Brilliant Blue G250. Ten microgram of protein was applied to each lane. M, prestained page ruler; lane 1, cell‐free extract; lane 2, cytoplasm; lane 3, pooled fractions from Q‐Sepharose; lane 4, pooled fractions from Phenyl‐Sepharose; lane 5, pooled fractions from Superdex 200.

Analyses of the purified enzyme separated on a 12% SDS/PAGE revealed one protein with an apparent molecular mass of 130 kDa (Fig. 2). Using peptide mass fingerprinting, this protein could be identified as the gene product of *pfor1* (TKV_c04340). Analytical size exclusion chromatography revealed a molecular mass of 245 kDa for the purified complex, which is consistent with PFOR1 being a homodimer.

### Biochemical characterization of PFOR1

**Fig. 3:**
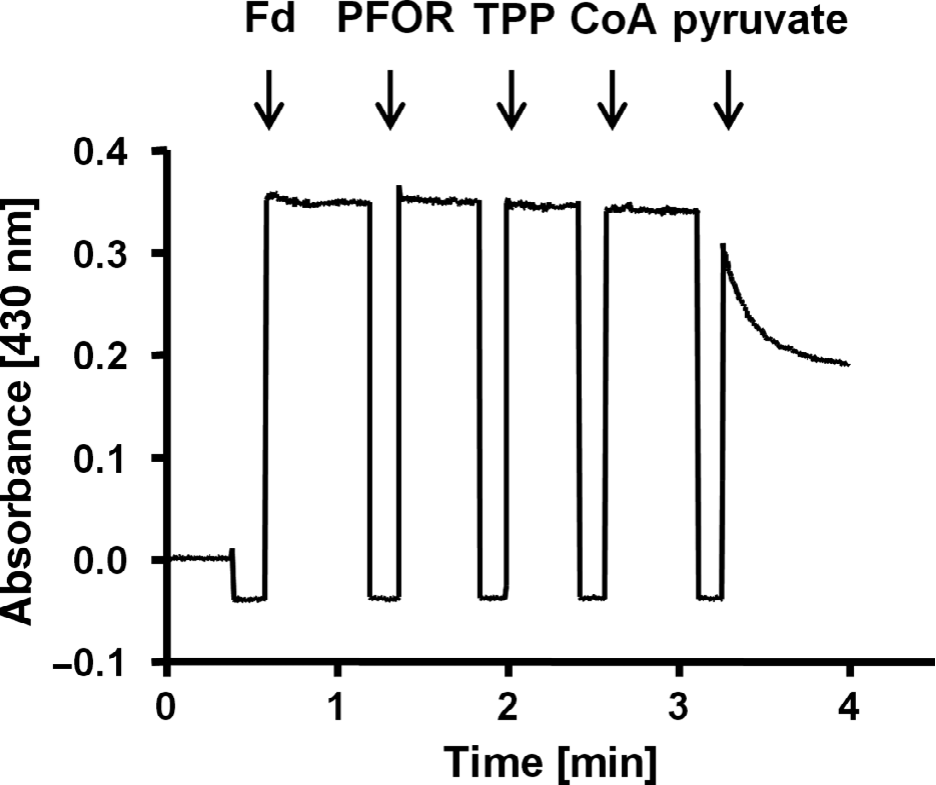
Pyruvate‐oxidizing activity of the purified PFOR1. Enzymatic activity was measured in 1.8‐mL anoxic cuvettes containing an overall liquid volume of 1 mL. The assay contained 5 μg PFOR, 200 μm CoA, and 50 μm TPP in buffer (50 mm Tris/HCl, 10 mm NaCl, 2 mm DTE, 4 μm resazurin, pH 7.5) under a 100% N_2_ atmosphere at 66 °C. 30 μm Fd served as electron acceptor. The reaction was started by addition of 10 mm pyruvate. Reduction of Fd was measured at 430 nm.

**Fig. 4:**
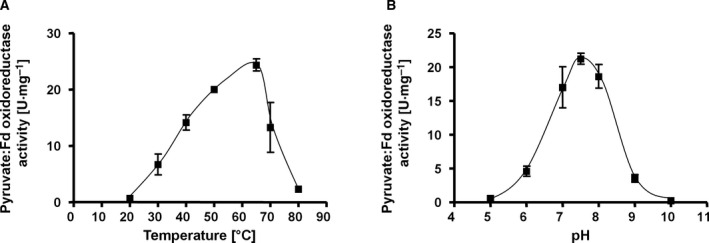
pH optimum and temperature profile of purified PFOR1. Temperature (A) or pH (B) dependence of the pyruvate‐dependent Fd reduction was measured in 1.8‐mL anoxic cuvettes containing an overall liquid volume of 1 mL under a 100% N_2_ atmosphere at 20–80 °C (A) or 66 °C (B). The assay contained 1 mL of buffer A (50 mm Tris/HCl, 10 mm NaCl, 2 mm DTE, 4 µm resazurin, pH 7.5) or buffer B (50 mm Tris, 50 mm MES, 50 mm CHES, 50 mm CAPS, 50 mm Bis/Tris, 10 mm NaCl, 2 mm DTE, 4 µm Resazurin, pH 5–10), 5 μg PFOR, 200 μm CoA, 50 μm TPP, 30 μm Fd and 10 mm pyruvate. Shown is the average of two measurements from one representative experiment out of two independent replicates. Error bars represent the SEM.

First, we assessed key biochemical properties of the purified PFOR1, including temperature and pH stability, substrate affinities, and cofactor dependence. To ensure an ideal reflection of the physiological conditions, we exclusively used the PFOR assay. The purified PFOR1 reduced Fd with pyruvate as reductant with an average specific activity of 27.2 ± 4.1 U·mg^−1^ (Fig. 3). PFOR1 was active at temperatures ranging from 22 to 80 °C with a maximal activity of 24.3 ± 1.1 U·mg^−1^ at the optimal growth temperature of *T*. *kivui* (66 °C) (Fig. 4A). The PFOR1 was not only active at mesophilic and thermophilic conditions but also extremely stable, with 70% activity remaining after 172 days of storage at 4 °C. The pH range was relatively narrow with only 20% activity at pH 6 and 8 and an optimal activity of 26.9 ± 0.4 at pH 7.5 (Fig. 4B). All further analyses were subsequently carried out at pH 7.5 and 66 °C, to ensure optimal enzyme activity.

Next, we assessed the K_m_ values for all reaction partners of the PFOR1. Since the enzyme was purified from cells grown on glucose, the physiological direction of the enzyme is to oxidize pyruvate to acetyl‐CoA, which is then further converted to acetate at the gain of ATP from substrate‐level phosphorylation. As mentioned, this PFOR activity of PFOR1 was 27.2 ± 4.1 U·mg^−1^. The dependence of the reaction on Fd, pyruvate, and CoA was hyperbolic with saturation at 10 mm pyruvate, 50 µm Fd, and 200 µm CoA (Fig. S1). The K_m_ values for pyruvate, Fd, and CoA were 0.11 ± 0.02 mm, 19.1 ± 3.4 µm, and 25.4 ± 4.7 µm, respectively (Fig. S1A–C). Unsurprisingly, the absence of any reaction partner led to a complete loss of activity.

### Cofactor determination of PFOR1

From bioinformatic analyses, it was inferred that PFOR1 should contain three iron–sulfur clusters ([4Fe–4S]) for electron transfer and no flavins (Fig. 1B). Indeed, we measured 13.6 ± 0.8 mol of iron·mol of protein^−1^ using the colorimetric assay to detect complexed iron [39], which matches the prediction. Flavins were not detected in the purified protein. Moreover, thiamine pyrophosphate (TPP) is a cofactor used by several PFOR enzymes [33, 40], because TPP acts as a potent nucleophile that forms an adduct with pyruvate and enzyme‐bound [4Fe–4S] clusters deliver electrons into or out of the active site [41]. A binding site for TPP was also detected in our *in silico* analyses for PFOR1 (Fig. 1B). And indeed, purified PFOR1 exhibited only a residual activity of 1.1 ± 0.2 U·mg^−1^ when TPP was omitted from the enzyme assay as opposed to 27.2 ± 4.1 U·mg^−1^ with 200 µm TPP in the assay. The dependence of the reaction on TPP was hyperbolic, reaching a saturation at 200 µm TPP, and the K_m_ value was 287.2 ± 0.1 nm (Fig. S1D).

### Rapid and simple production of genetically modified PFOR1 in *T. kivui*

**Fig. 5:**
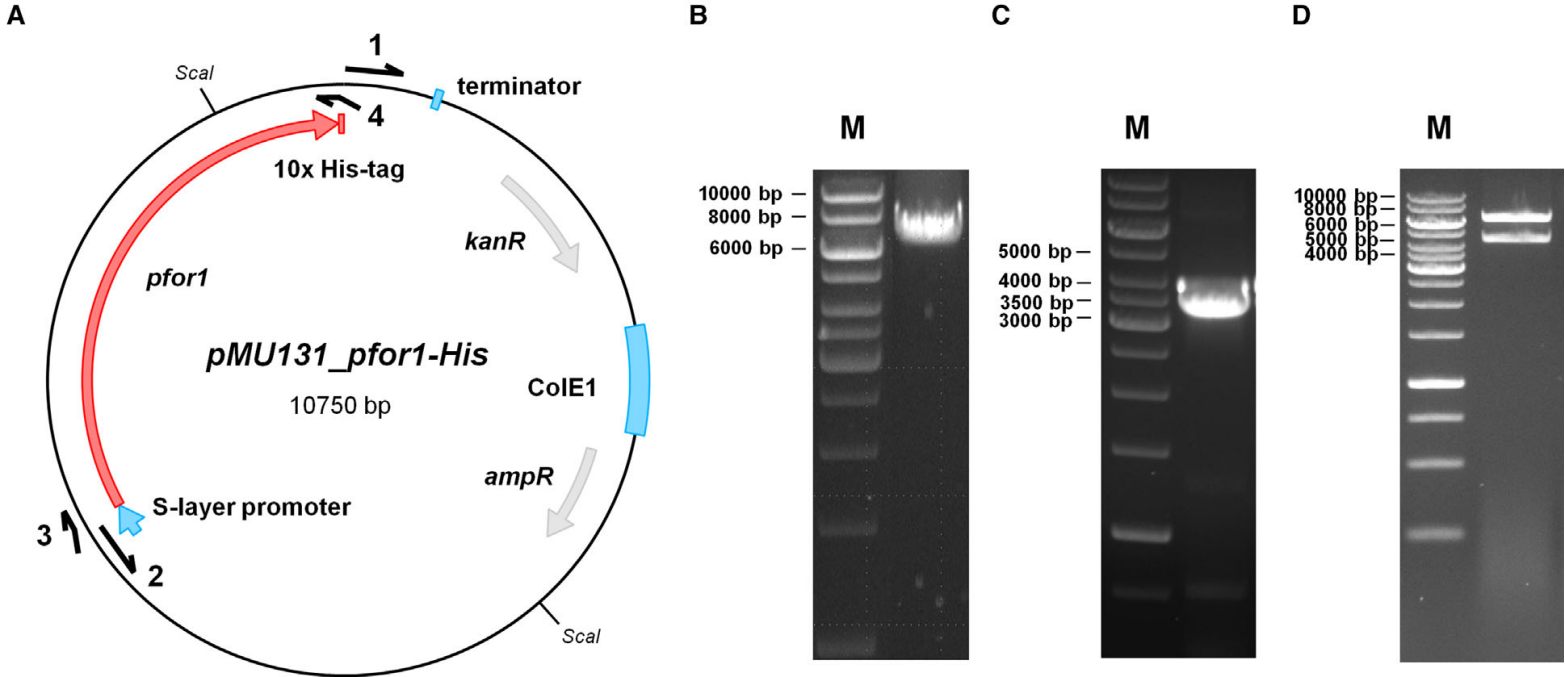
Cloning of *pMU131_pfor1‐His*. For the production of PFOR1‐His in *Thermoanaerobacter kivui,* the construct *pMU131_pfor1‐His* was cloned (A). Therefore, *pMU131* backbone, including a S‐Layer promoter, was amplified using corresponding primers pMU131_for (1) and pMU131_rev (2) via PCR (size: 7192 bp) (B). *Pfor1* was amplified from genomic DNA of *T. kivui* via PCR, using PFOR1‐His_for (3) and PFOR1‐His_rev (4) primers (size: 3598 bp) (C). PFOR1‐His_rev primer contained an additional DNA sequence coding for a 10x His‐tag. Amplified *pfor1‐His* and *pMU131* backbone were fused via Gibson Assembly and transformed in *E. coli* HB101. Afterward, plasmids were isolated and digested with *Sca*I (D). The resulting sizes were 4241 bp and 3407 bp. M, Gene Ruler 1 kb DNA ladder.

**Fig. 6:**
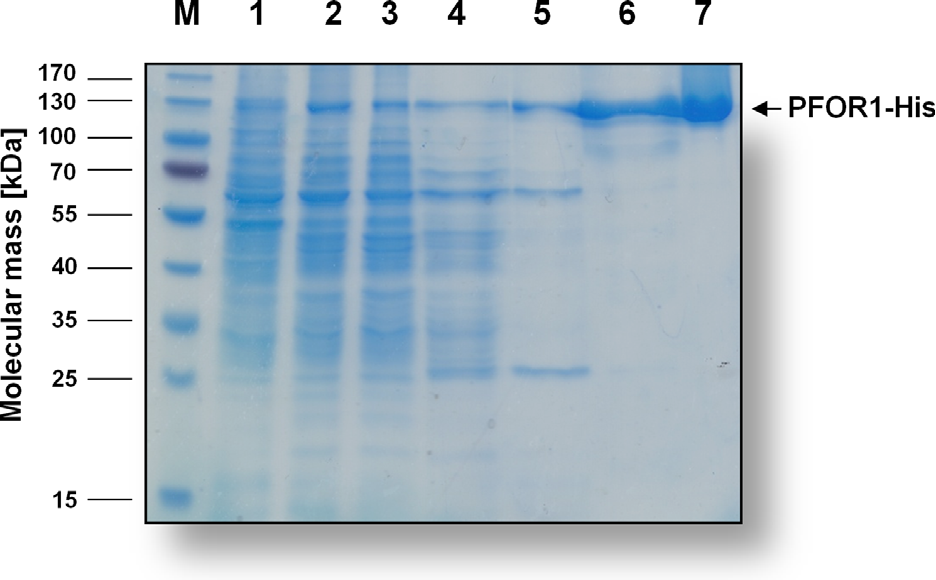
SDS/PAGE monitoring the purification process of PFOR1‐His. Samples of the different purification steps were separated by SDS/PAGE (12%) and proteins were stained with Coomassie Brilliant Blue G250. Ten microgram of protein was applied to each lane. M, prestained page ruler; lane 1, *T. kivui* cells; lane 2, cell‐free extract; lane 3, flow through; lane 4, wash fraction; lane 6, pooled Ni^2+^‐NTA elution fractions; lane 7, pooled size exclusion fractions.

To increase the yield and simplify the purification of PFOR1, we took advantage of a plasmid, *pMU131*, which is replicating in *T*. *kivui* [42]. The plasmid has already been used for gene expression in *T. kivui* to complement growth phenotypes. Among others, *T. kivui* phosphofructokinase *fruK* in a ∆*fruK* background proving the involvement of *fruK* in fructose metabolism [42]. Here, we aimed for overproduction of PFOR1. Therefore, we cloned the *pfor1* gene (TKV_c04340) together with a gene sequence coding for a 10x histidine‐tag into a plasmid containing the S‐layer promoter of *T. kivui* (Fig. 5). Naturally competent cells of *T. kivui* were transformed with the verified plasmid (Fig. S2) and cell‐free extract of the genetically modified *T. kivui* strain was prepared as described for the wild type. Undisrupted cells were removed by centrifugation and the His‐tagged PFOR1 was purified from the cell‐free extract on Ni^2+^‐NTA‐Sepharose followed by a size exclusion chromatography on Superdex 200. This procedure yielded an apparently homogeneous preparation with just two purification steps (Fig. 6). 5.4 mg of the enzyme was purified 36‐fold to apparent homogeneity out of a 1 l culture (Table 2). The purified tagged version exhibited almost similar average specific activity of 21.8 ± 2.3 U·mg^−1^ as the untagged version. Therefore, the tag did not interfere with PFOR1 activity. Using this protocol, it was possible to obtain fully functional PFOR1 with a 135 times higher yield in one rather than 4 days.

**Table 2:** Purification of PFOR1‐His from *Thermoanaerobacter kivui*.

Purification step	Protein (mg)	PFOR activity^a^ (U)	PFOR activity (U·mg^−1^)	Purification (‐fold)	Yield (%)
Cell‐free extract	193	120.2	0.59	1	100
Ni^2+^‐NTA	6.5	114	17.5	30	94.8
Superdex 200	5.4	112.9	21.1	35.8	93.9

^a^ PFOR activity was measured with pyruvate as electron donor and ferredoxin as electron acceptor.

## Discussion

In this work, we discovered, purified, and characterized a very stable PFOR from the anaerobic acetogenic bacterium *T. kivui*. Moreover, we developed an improved and simple purification protocol for PFOR, based on homologous overproduction in a strict anaerobe, a method that may be transferred to other strictly oxygen‐sensitive proteins.

PFOR1 is the enzyme in *T. kivui* that provides acetyl‐CoA and reduced Fd from pyruvate during heterotrophic growth. Under heterotrophic conditions, the function of PFOR1 is to connect glycolysis with acetate production. The two electrons that are generated as a result of pyruvate decarboxylation are used in the reduction of low‐potential ferredoxins (Fd^2−^), which are used to fuel the chemiosmotic gradient by the respiratory Ech‐complex [21] and the reduction of CO_2_ to protein‐bound CO by the CODH/ACS [43] in the WLP.

*In silico* analysis using ‘InterPro’ [44] revealed that PFOR1 belongs to the 2‐oxoacid:ferredoxin oxidoreductase (OFOR) superfamily and has a typical six‐domain arrangement (Fig. 1B) [45]. PFOR1 is 65% or 61% identical to well‐studied PFORs of *Moorella thermoacetica* (MtOOR) [41] or *Desulvovibrio africanus* (DaOOR) [46], respectively. The N‐terminal region comprises a pyruvate flavodoxin/ferredoxin oxidoreductase (domain I) that is involved in TPP‐binding, followed by a second domain II, which might be involved in interaction with another protein subunit. In the middle of the protein is the domain III (gamma domain). Recently, a crystal structure of a PFOR from *M*. *thermoacetica* (MtOOR) revealed that residues of domain III form a phosphate‐binding loop (P‐loop) that is responsible for CoA binding [41]. The same P‐loop for CoA binding is conserved in PFOR1 of *T. kivui*. Next, a 33‐residue‐long conserved domain containing an EKR sequence motif (domain IV) follows. It has a hitherto unknown function and links the PFOR1 domain to a following Fe‐S‐cluster binding domain (domain V) with a C‐X2‐C‐X2‐C‐X3‐C binding motif. Finally, at the C terminus is a TPP‐ and pyruvate‐binding domain (domain VI). In PFOR1, the N‐ and C‐terminal region contains a thiamine diphosphate‐binding fold, comprising two functional modules: the pyridine‐binding and pyrophosphate‐binding module. Two of a kind can assemble, giving rise to a homodimer with a heterotetramic core that binds two thiamine diphosphate molecules [32]. The burial of TPP in the dimerized PFOR explains why activity was still present (although lower) in assays containing enriched PFOR1 from *T. kivui* without TPP supplementation.

Besides PFOR1, the genome of *T. kivui* encodes for a 73% identical second PFOR with an exact similar six‐domain arrangement (PFOR2; Fig. 1B). Surprisingly, after the purification from glucose‐grown cells, there were no indications that PFOR2 was copurified. This could be explained by an absence of the protein due to no or very low gene expression, or a different activity of this enzyme. Indeed, the occurrence of multiple PFOR‐encoding genes is also found in several other organisms like *Thermoanaerobacterium saccharolyticum* [47], *M*. *thermoacetica* [41, 48], or *Pyroccocus furiosus* [33, 49]. While *pfor1* in *T. kivui* encodes the catalytic PFOR1, the other presumably encodes a similar enzyme with another substrate specificity (possibly to another α‐ketoacid). Alternatively, it might also convert pyruvate, but use an alternate electron acceptor. Good candidates for this could be flavodoxin, rubredoxin, or thioredoxin [50], which may substitute the role of Fd under iron deprivation. After all, not much is known about these electron carriers in acetogens.

In this work, we established a simple and rapid purification method for PFOR1 from *T. kivui* that can be used as ‘supporting‐enzyme’ for biochemical analysis in the future. The ability to reduce Fd (even from *C. pasteurianum*) at moderate pH values, and mesophilic and thermophilic conditions makes the PFOR1 of *T. kivui* an invaluable enzyme during *in vitro* studies. Moreover, the enzyme is stable for a long time. Reduced Fd is not only an electron donor for many electron‐bifurcating enzymes [51], but is also required for the acetogenic respiratory enzymes, like Ech complexes [21] or Rnf complexes [22]. PFOR1 of *T. kivui* catalyzes the oxidation of pyruvate to acetyl‐CoA and CO_2_ (E_0_^′^ = −500 mV) [15, 16] coupled to the reduction of Fd (E_0_'[Fd^2−^/Fd] ~ −450 to −500 mV) [16] as electron donor. Since this system does not involve strong chemical reducing agents such as sodium dithionite or titanium (III) citrate, which often interfere with the physiological reaction of several enzymes [21, 22, 52], it is an ideal way to provide reduced Fd for biochemical assays in a physiological and nontoxic manner.

## Methods

### Growth of cells and purification of the PFOR

*T. kivui* (DSM 2030) was grown at 66 °C under anoxic conditions in 20‐L bottles (Glasgerätebau Ochs, Bovenden‐Lenglern, Germany) using 28 mm d‐glucose as substrate. The medium and all buffers were prepared using the anaerobic techniques described previously [31, 53, 54]. All buffers used for preparation of cell extracts and purification contained 2 mm DTE, 4 µm resazurin, and 20% [v/v] glycerol. All purification steps were performed under strictly anaerobic conditions at room temperature in an anaerobic chamber (Coy Laboratory Products, Grass Lake, MI, USA) filled with 95–98% N_2_ and 2–5% H_2_. Cells of *T. kivui* were harvested and washed twice in buffer A (50 mm Tris/HCl, 2 mm DTE, 4 µm resazurin, 20% (v/v) glycerol, pH 8.0). The cells were resuspended in 50 mL buffer A including 0.5 mm PMSF and 0.1 mg·mL^−1^ DNAseI and passed two times through a French pressure cell (110 MPa). Cell debris was removed from the cell‐free extract by centrifugation at 24 000 ***g***, 4 °C for 20 min. Membranes were removed by centrifugation at 130 000 ***g***, 4 °C for 60 min. The supernatant containing the cytoplasmic fraction was diluted with 150 mL buffer A and applied to a Q‐Sepharose high‐performance column (2.6 cm × 40 cm, 31 mL Q‐Sepharose) equilibrated with buffer A. Protein was eluted with a linear gradient of 250 mL from 0 to 1 m NaCl in buffer B (50 mm Tris/HCl, 1 m NaCl, 20 mm MgSO_4_, 2 mm DTE, 4 µm resazurin, 20% (v/v) glycerol, pH 8.0). PFOR activity eluted at around 3–13 mm NaCl or conductivity of 3.8–8.4 mS·cm^−1^. Ammonium sulfate (1 m) was added to the pooled fractions and these were loaded onto a Phenyl‐Sepharose high‐performance column (1.6 cm × 10 cm, 27 mL Phenyl‐Sepharose) equilibrated with buffer C (50 mm Tris/HCl, 20 mm MgSO_4_, 1 m (NH_4_)_2_SO_4_, 2 mm DTE, 4 µm resazurin, 20% (v/v) glycerol, pH 7.5). Protein was eluted with a linear gradient of 170 mL from 1 to 0 m (NH_4_)_2_SO_4_ in buffer D (50 mm Tris/HCl, 20 mm MgSO_4_, 2 mm DTE, 4 µm resazurin, 20% (v/v) glycerol, pH 7.5). PFOR activity eluted in a peak around 0.84–0.68 m (NH_4_)_2_SO_4_ or conductivity of 64–55 mS·cm^−1^. Pooled fractions were concentrated using ultrafiltration in 50‐kDa VIVASPIN tubes (Sartorius Stedim Biotech GmbH, Germany). The sample was loaded on a Superdex 200 increase 10/300 GL (GE Healthcare Life Sciences, Little Chalfont, UK) equilibrated with buffer E (50 mm Tris/HCl, 150 mm NaCl, 20 mm MgSO_4_, 2 mm DTE, 4 µm resazurin, 20% (v/v) glycerol, pH 7.5) and eluted at a flow rate of 0.5 mL·min^−1^. PFOR activity eluted in a single peak with a maximum at 12.5 mL elution volume. Fractions corresponding to this peak were pooled and stored in buffer E at 4 °C.

### Cloning of *pMU131_pfor1‐His*

Plasmid *pMU131_pfor1‐His* was used for the expression of *pfor1* (TKV_c04340). The plasmid is based on plasmid *pMU131* [55] which is replicating in *T. kivui* and confers resistance to kanamycin [42]. The insert was amplified by using the primers PFOR1_His_for (5′‐CAA GGA GGA GGA TTG ACT GTA TGG CTA AGG TAA TGA AG‐3′) and PFOR1_His_rev (5′‐TCC TGG ATA AAT TTA AAA AAT TAA TGA TGA TGA TGA TGG TGA TGA TGA TGG TGT TCA TCT TTT GCT AAT TTT TCG TAG‐3′). The backbone *pMU131* was amplified by using the primers pMU131_for (5′‐TTT TTT AAA TTT ATC CAG GAT AAA AGA GAA GAC TC‐3′) and pMU131_rev (5′‐ACA GTC AAT CCT CCT CCT TG‐3′), followed by the fusion of the PCR products via Gibson Assembly. *T. kivui* (DSM 2030) was transformed with the generated plasmid *pMU131_pfor1‐His*, taking advantage of its natural competence for DNA uptake [42]. Following the transformation protocol of Basen *et al*. [42], cells were plated on agar medium using 28 mm glucose as carbon source and 200 µg·mL^−1^ kanamycin as selection marker. To verify the transformation, colonies were picked and the transformed plasmids were checked by using primer pairs seq1_for (5′‐TCT AAC ACA ATT ATA TCA TAA GGA TTG ATA‐3′)/seq2_rev (5′‐AGT ATT GTC AAT ATA TTC AAG GCA A‐3′) binding on the *pMU131* backbone and amplifying the complete *pfor1* locus.

### Production and purification of His‐tagged PFOR1 in *T. kivui*

For the purification of the His‐tagged PFOR1, *T. kivui pMU131_pfor1‐His* cells were grown in the presence of 28 mm glucose and 200 µg·mL^−1^ kanamycin. The preparation of cell‐free extract was carried out as described previously, using a modified buffer A (50 mm Tris/HCl, 150 mm NaCl, 20 mm MgSO_4_, 10 mm imidazole, 0.5 mm DTE, 4 µm resazurin, 20% (v/v) glycerol, pH 7.5). Protein purification was carried out on a nickel nitrilotriacetic acid (Ni^2+^‐NTA) resin (Qiagen, Hilden, Germany) using a gravity flow column under anoxic conditions. Cell‐free extract was incubated with 1 mL resin for 10 min at room temperature. Afterward, the resin was washed with buffer F (50 mm Tris/HCl, 150 mm NaCl, 20 mm MgSO_4_, 30 mm imidazole, 0.5 mm DTE, 4 µm resazurin, 20% (v/v) glycerol, pH 7.5) to remove loosely bound proteins from the resin. Subsequently, specifically bound proteins were eluted by adding 400 mm imidazole‐containing elution buffer G (50 mm Tris/HCl, 150 mm NaCl, 20 mm MgSO_4_, 400 mm imidazole, 0.5 mm DTE, 4 µm resazurin, 20% (v/v) glycerol, pH 7.5). One milliliter fractions were collected, pooled, concentrated, using 50‐kDa VIVASPIN tubes, and separated on a Superdex 200 increase 10/300 GL (GE Healthcare Life Sciences) as described above. Fractions containing PFOR1‐His were pooled and stored at 4 °C.

### Measurement of PFOR enzyme activity

Enzyme assays were routinely performed at 66 °C in 1.8‐mL anoxic cuvettes (Glasgerätebau Ochs, Bovenden‐Lenglern, Germany) sealed by rubber stoppers in a N_2_ atmosphere with buffer H (50 mm Tris/HCl, 10 mm NaCl, 4 mm DTE, 4 μm resazurin, pH 7.5) at an overall liquid volume of 1 mL. PFOR activity was measured with MB or Fd as electron acceptor and measured at 665 nm (ε = 53.1 mm ^−1^·cm^−1^) or 430 nm (ε = 13.1 mm ^−1^·cm^−1^), respectively. Fd was purified from *C. pasteurianum* as described previously [56]. The assay was supplemented with cell‐free extract, cytoplasm or enriched PFOR preparations, 50 µm MB or 30 μm Fd, 200 μm CoA, and 100 μm TPP. The reaction was started by addition of 10 mm sodium pyruvate. For *K*_m_ determination, the CoA, pyruvate, TPP and Fd concentrations ranged between 0–200 µm, 0–10 mm, 0–20 µm, and 0–50 µm, respectively. For the determination of the pH and temperature profile, the assay and protein were preincubated for 10 min at the pH or temperature indicated. The buffer used for the pH optima determination was 50 mm MES, 50 mm CHES, 50 mm CAPS, 50 mm Bis/Tris, 50 mm Tris, 10 mm NaCl, 4 mm DTE, 4 μm resazurin at pH 5–10.

### Analytical methods

The concentration of proteins was measured according to Bradford [57]. Proteins were separated in 12% SDS/PAGE and stained with Coomassie Brilliant Blue G250. The molecular mass of the purified PFOR was determined using a calibrated Superdex 200 column, buffer E, and defined size standards (ovalbumin: 43 kDa; albumin: 158 kDa; catalase: 232 kDa; ferritin: 440 kDa). The isolated PFOR was identified by matrix‐assisted laser desorption/ionization‐time of fight (MALDI‐TOF) analysis. Peptide mass fingerprinting by MALDI‐TOF analysis was performed by the ‘Functional Genomics Center Zürich’ at the ETH Zurich, Switzerland, and results were analyzed using the Scaffold‐Proteome Software version 4.10.0 (Proteome Software Inc., Portland, OR, USA). The iron content of the purified enzyme was determined by colorimetric methods [39]. Flavin determination was performed by TLC as described before [25].

## Conflict of interest

The authors declare no conflict of interest.

## Author contributions

VM, AK, MCS, and MB designed the experiments. AK and MCS performed the experiments. AK, MCS, MB, and VM wrote the paper.

## Supporting information


**Fig. S1.** Ferredoxin, pyruvate, CoA and TTP dependence on PFOR1 activity. PFOR activity was measured in 1.8‐mL anoxic cuvettes containing an overall liquid volume of 1 mL under a 100% N_2_ atmosphere at 66 °C. The assay contained 1 mL of buffer A (50 mm Tris/HCl, 10 mm NaCl, 2 mm DTE, 4 µm resazurin, pH 7.5), 5 μg PFOR, different amounts of pyruvate (A), Fd (B), CoA (C), or TPP (D). Shown is the average of two measurements from one representative experiment out of two independent replicates. Error bars represent the SEM.

**Fig. S2.** Verification of the *pMU131_pfor1‐His* construct transformed in *Thermoanaerobacter kivui*. To verify the nature of the plasmid *pMU131_pfor1‐His* after propagation, *T. kivui* colonies were picked and the plasmid was checked by using primer pairs seq1_for (5)/ seq2_rev (6) binding on the *pMU131* backbone and amplifying the complete *pfor1‐His* (A). The resulting size was 4054 (B). M, Gene Ruler 1 kb DNA ladder.

## Data Availability

The data that support the findings of this study are available from the corresponding author (vmueller@bio.uni-frankfurt.de) upon reasonable request.
