# Supplementary figures and images for: The pyruvate:ferredoxin oxidoreductase of the thermophilic acetogen, Thermoanaerobacter kivui
Source: FEBS Open Bio. 2021 Apr 4;11(5):1332–42. doi: 10.1002/2211-5463.13136 (PMC8091585; doi:10.1002/2211-5463.13136)

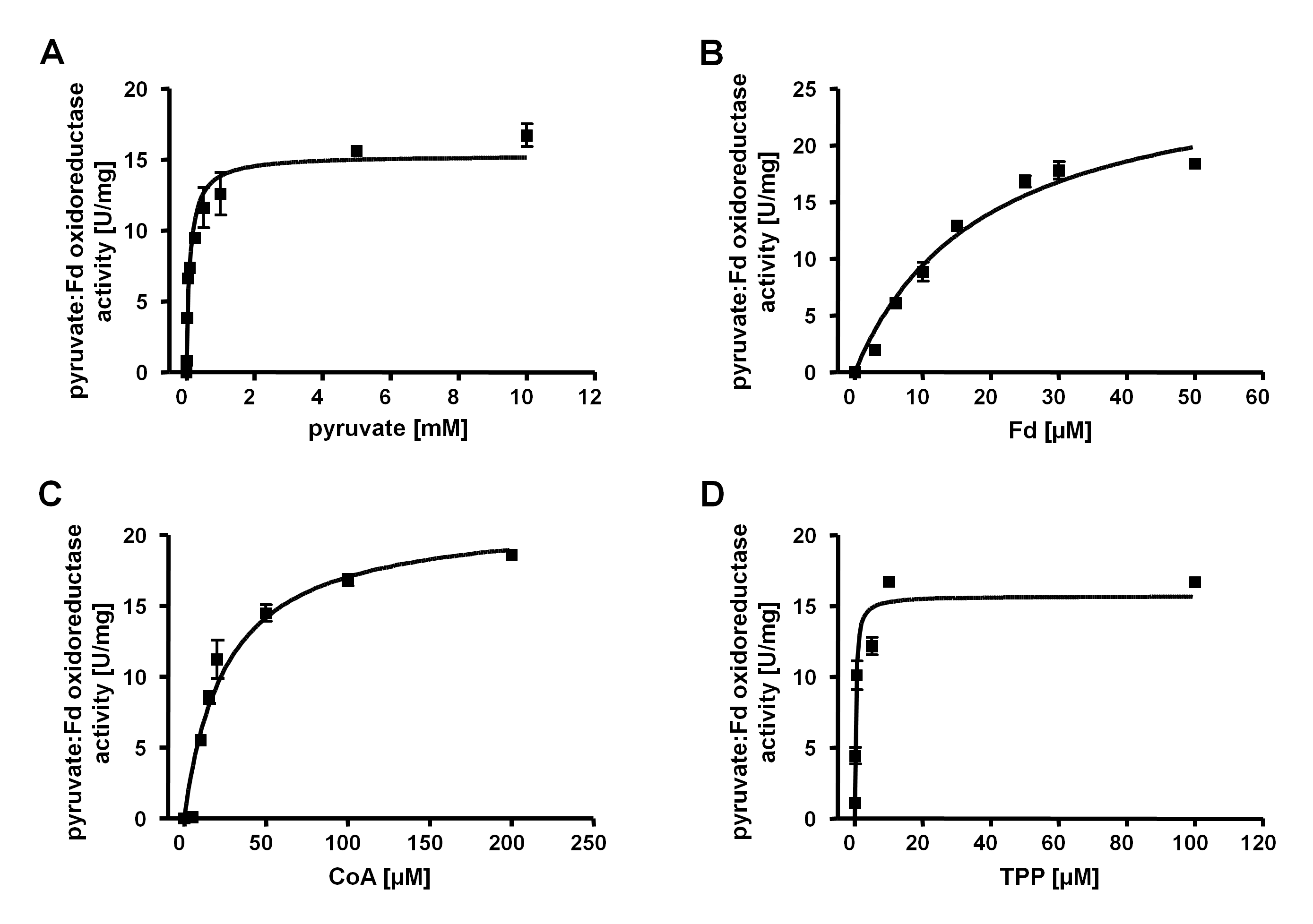

Supplement: Supplementary file 1 — Fig. S1. Ferredoxin, pyruvate, CoA and TTP dependence on PFOR1 activity. PFOR activity was measured in 1.8‐mL anoxic cuvettes containing an overall liquid volume of 1 mL under a 100% N2 atmosphere at 66 °C. The assay contained 1 mL of buffer A (50 mm Tris/HCl, 10 mm NaCl, 2 mm DTE, 4 µm resazurin, pH 7.5), 5 μg PFOR, different amounts of pyruvate (A), Fd (B), CoA (C), or TPP (D). Shown is the average of two measurements from one representative experiment out of two independent replicates. Error bars represent the SEM. [file FEB4-11-1332-s001.tif]

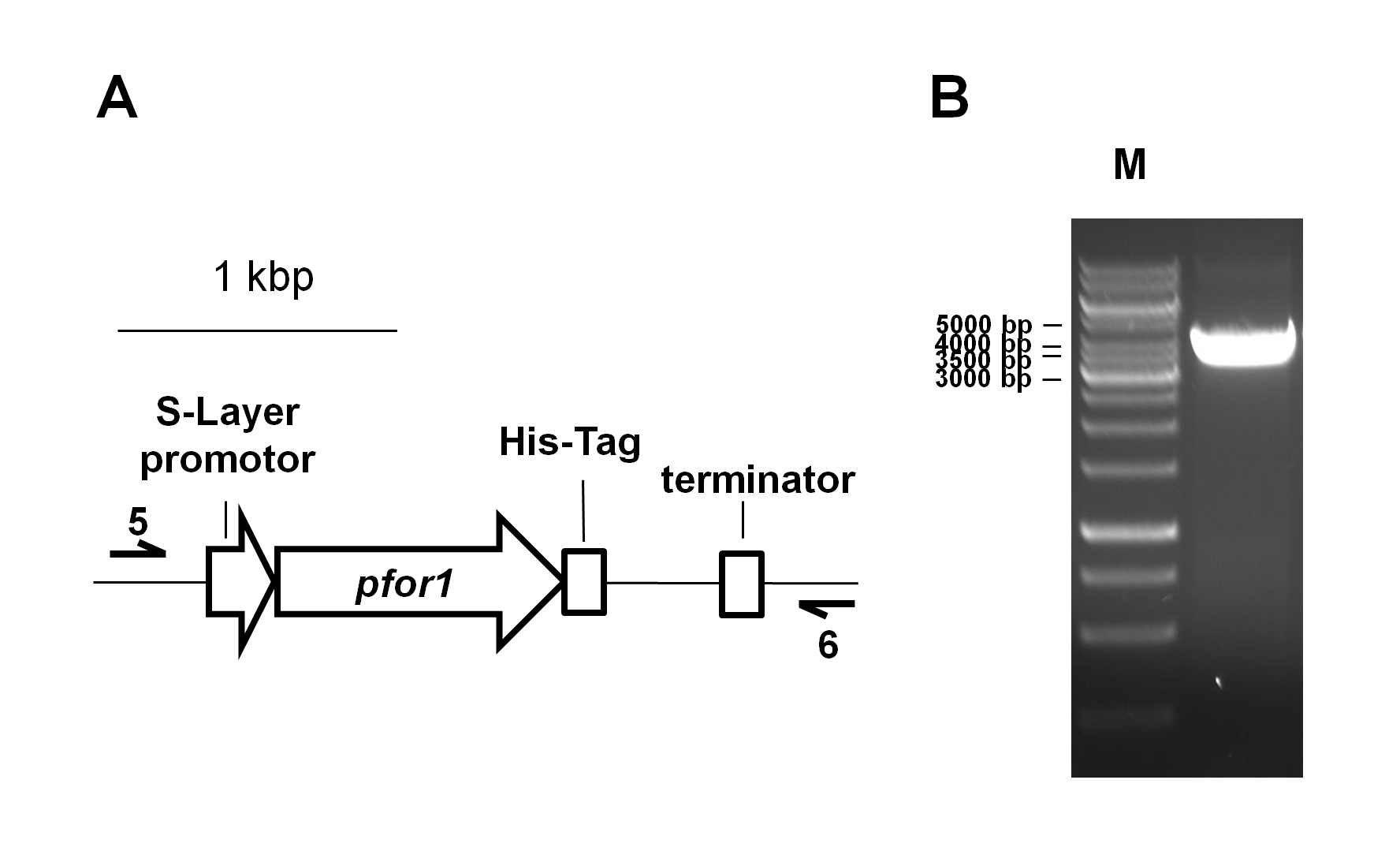

Supplement: Supplementary file 2 — Fig. S2. Verification of the pMU131_pfor1‐His construct transformed in Thermoanaerobacter kivui. To verify the nature of the plasmid pMU131_pfor1‐His after propagation, T. kivui colonies were picked and the plasmid was checked by using primer pairs seq1_for (5)/ seq2_rev (6) binding on the pMU131 backbone and amplifying the complete pfor1‐His (A). The resulting size was 4054 (B). M, Gene Ruler 1 kb DNA ladder. [file FEB4-11-1332-s002.tif]
